# Molecular epidemiological analysis of wild animal rabies isolates from India

**DOI:** 10.14202/vetworld.2019.352-357

**Published:** 2019-03-04

**Authors:** Gundallhalli Bayyappa Manjunatha Reddy, Rajendra Singh, Karam Pal Singh, Anil Kumar Sharma, Sobharani Vineetha, Mani Saminathan, Basavaraj Sajjanar

**Affiliations:** 1 Department of Veterinary Pathology, ICAR-National Institute of Veterinary Epidemiology and Disease Informatics, Bengaluru, Karnataka, India; 2 Department of Veterinary Pathology, ICAR-Indian Veterinary Research Institute, Bareilly, Uttar Pradesh, India

**Keywords:** India, nucleoprotein gene, phylogenetic analysis, rabies virus, wild animals

## Abstract

**Aim:**

This study was conducted to know the genetic variability of rabies viruses (RVs) from wild animals in India.

**Materials and Methods:**

A total of 20 rabies suspected brain samples of wild animals from different states of India were included in the study. The samples were subjected for direct fluorescent antibody test (dFAT), reverse transcription polymerase chain reaction (RT-PCR), and quantitative reverse transcriptase real-time PCR (RT-qPCR). The phylogenetic analysis of partial nucleoprotein gene sequences was performed.

**Results:**

Of 20 samples, 11, 10, and 12 cases were found positive by dFAT, RT-PCR, and RT-qPCR, respectively. Phylogenetic analysis showed that all Indian wild RVs isolates belonged to classical genotype 1 of Lyssavirus and were closely related to Arctic/Arctic-like single cluster indicating the possibility of a spillover of rabies among different species.

**Conclusion:**

The results indicated the circulation of similar RVs in sylvatic and urban cycles in India. However, understanding the role of wild animals as reservoir host needs to be studied in India.

## Introduction

Rabies is enzootic and is a serious public health and economic problem in India. Rabies virus (RV) belongs to phylogroup I of genus *Lyssavirus* and family *Rhabdoviridae*. Of the 14 distinct species recognized within the genus *Lyssavirus*, the classical RV is the most prevalent and widely distributed [1]. Dog-mediated rabies account to nearly 20,000 human deaths per year in India and are considered as principal reservoir hosts followed by cats and other wild animals [2,3]. Recently, 21 cases of human rabies have been reported in Edakkad, Kannur district, Kerala, India, due to mongoose bite. This is the first report from India on a massive attack of humans by mongoose and rabies transmission [4].

The transmission of rabies from wild animals is rarely reported in endemic countries such as India [2,3,5]. The occurrence of dog rabies has masked the importance of wild animal rabies in India. In rabies control programs, more attention was given to dog-mediated rabies, but there is a need to monitor for possible risk of wildlife populations getting infected with rabies. The genetic characterization of urban RABV isolates from domestic and human origin has been well documented by targeting different regions of RV genome [6-8].

However, sylvatic or wild animal rabies in India is not studied extensively, and the reports are scanty [3,9]. Therefore, an attempt was made on limited non-random rabies suspected brain samples to understand the genetic related of wild animal RVs with other domestic animals in India.

## Materials and Methods

### Ethical approval

No live wild animals were used during the study. Suspected brain samples from different species of wild animals that were already dead were submitted for laboratory confirmation of rabies.

### Study area and rabies isolates

**Figure-1:**
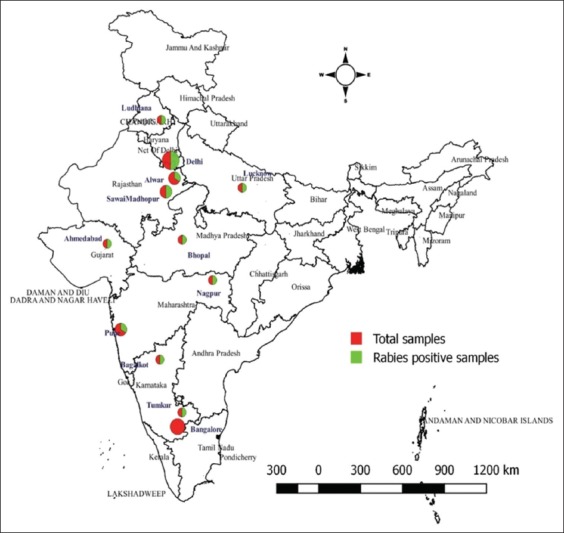
The map shows 20 rabies suspected sample collected locations that were surveyed in 2007-2017 (Source: The map was generated with the help of QGIS 2.18 software).

Between 2007 and 2017, 20 rabies suspected brain samples from dead animals covering different species of wild animals from different states of India such as Delhi (sambar deer, Himalayan sloth bear, hyena, and mongoose), Gujarat (hyena), Karnataka (wolf and bear), Punjab (jackal), Rajasthan (hyena), and Uttar Pradesh (bear) were submitted for confirmatory rabies diagnosis (Table-1 and Figure-1). Among them, 17 brain samples were received over ice and three samples in 10% neutral buffered formalin (NBF) as a preservative. The samples were processed for direct fluorescent antibody test (dFAT), reverse transcription-polymerase chain reaction (RT-PCR), quantitative reverse transcriptase real-time PCR (RT-qPCR), and histopathology.

**Table-1:** Sample details, diagnosis (dFAT+qRT-PCR), and GenBank accession ID of nucleotide sequences of different wild animal species from India.

Wild animal species	Number of samples	Place of sample collection	State	Result	Accession number
Bear	4	Tumakuru	Karnataka	+	KT381855
		Delhi	Delhi	+	KX685267
		Lucknow	Uttar Pradesh	+	GU371904
		Pune	Maharashtra	+*	-
Hyena	6	Alwar	Rajasthan	+	GU371899
		Ahmadabad	Gujarat	+	GU371876
		Delhi	Delhi	+	KX685268
		Sawai Madhopur	Rajasthan	+	Could not amplify by conventional
		Sawai Madhopur	Rajasthan	+	PCR (+by real-time PCR)
		Alwar	Rajasthan	−	-
Jackal	2	Ludhiana	Punjab	+	MG181948
		Bhopal	Madhya Pradesh	+*	-
Leopard	1	Bengaluru	Karnataka	−	-
Mongoose	1	Delhi	Delhi	+	KX685269
Sambar deer	1	Delhi	Delhi	+	KX685270
Wolf	3	Bagalkot	Karnataka	+	KT381854
		Nagpur	Maharashtra	+*	-
		Pune	Maharashtra	−	-
Monkey	1	Bengaluru	Karnataka	−	-
Civet cat	1	Bengaluru	Karnataka	−	-

* Histopathology only, dFAT=Direct fluorescent antibody test, qRT=Quantitative real time, PCR=Polymerase chain reaction

### dFAT

The RV antigen in the brain impression smears was detected by dFAT as recommended by the World Health Organization [10]. Briefly, impression smears from coronal sections of suspected brain samples were prepared and were fixed with chilled acetone. Before staining, the slides were given three washes with 1X phosphate-buffered saline (PBS, pH 7.2) for 5 min each followed by incubation with anti-rabies FITC conjugate (Millipore, USA) in a humidified dark chamber at 37°C for 1 h. The slides were washed thoroughly in PBS, mounted with VECTASHIELD Antifade Mounting Medium (Vector Laboratories, CA, USA), and examined under a fluorescent microscope (Nikon, Japan). During each run, positive and negative controls were run along with test slides.

### Viral RNA isolation

Viral RNA from approximately 100 mg brain tissues was isolated using QIAamp Viral RNA Mini Kit (QIAGEN, Hilden, Germany), following the manufacturer’s instructions, and quantified using spectrophotometer (ND-2000, Thermo Scientific, USA).

### cDNA synthesis and RT-PCR

Complementary DNA (cDNA) was synthesized from the viral RNA by QuantiTect Reverse Transcription Kit (QIAGEN, Hilden, Germany), following the manufacturers’ protocol. cDNA was prepared from approximately 1 μg of viral RNA by incubating 20 μl reaction mixture at 42°C for 30 min followed by heat inactivation at 95°C for 3 min. RT-PCR was performed using N gene-specific primers [7] in 50 µl reaction mixture containing Platinum Pfx DNA Polymerase (2.5 U/µl) using gradient thermocycler (Mastercycler Personal, Eppendorf, Germany). The PCR products were electrophoresed, and gel-containing specific amplicons were sliced and were subjected for gel extraction using QIAquick Gel Extraction Kit (QIAGEN, Hilden, Germany).

### Quantitative real-time (qRT)-PCR

Irrespective of dFAT and RT-PCR results, all the samples were subjected for SYBR green dye-based RT-qPCR using specific primers [7] in MX3000P quantitative PCR System (Stratagene, USA). A melting curve analysis was performed to know the specificity of RT-qPCR.

### Histopathology

Three 10% NBF preserved brain tissues were processed for histopathological examination. Tissue samples of 1-2 mm thickness were dehydrated in graded alcohol and cleared in xylene and embedded in paraffin blocks. The 4-5 μ thick sections were taken with rotator microtome on clean grease-free slides and subjected for hematoxylin and eosin staining. The brain tissue sections were examined under a light microscope for the presence of characteristic eosinophilic intracytoplasmic Negri bodies.

### Nucleotide sequencing and phylogenetic analysis

The paired-end Sanger’s dideoxy sequencing (Xcelris Genomics, India) was performed with forward and reverse N gene primers. The edited sequences were submitted to GenBank and were assigned accession numbers (Table-1). The sequence alignment was performed in muscle program, and phylogenetic tree was derived at 1000 bootstraps by maximum likelihood method based on the Tamura–Nei model in MEGA7.0 [11]. Published N gene sequences from India and rest of the world were also included for phylogenetic analysis (supplementary file).

## Results

### Detection of RV

Impression smears from rabies-positive brain tissues showed characteristic diffuse apple green fluorescent signals of RABV antigen with varying degrees in the cytoplasm of the neurons by dFAT. Of 20 rabies suspected samples, 11 samples were found positive with dFAT. The RNA was isolated from 17 brain samples, which were received over ice. However, RNA could not be isolated from three formalin-fixed samples. Of 17 samples, conventional RT-PCR amplified the N gene of RV with an expected product size of 806 bp in 10 samples, and 12 samples were positive by RT-qPCR without any non-specific reactions with specific melting temperature (82.24-83.11°C) (Table-1).

### Histopathology

Three formalin-fixed brain samples of wild animals were positive for rabies by revealing characteristic round-to-oval, eosinophilic intracytoplasmic Negri bodies in the pyramidal cells of the hippocampus and cerebellar Purkinje cells. Other histopathological changes were neuronal necrosis, satellitosis, gliosis, neuronophagia, congestion, edema, perivascular cuffing, and meningitis.

### Phylogenetic analysis

**Figure-2:**
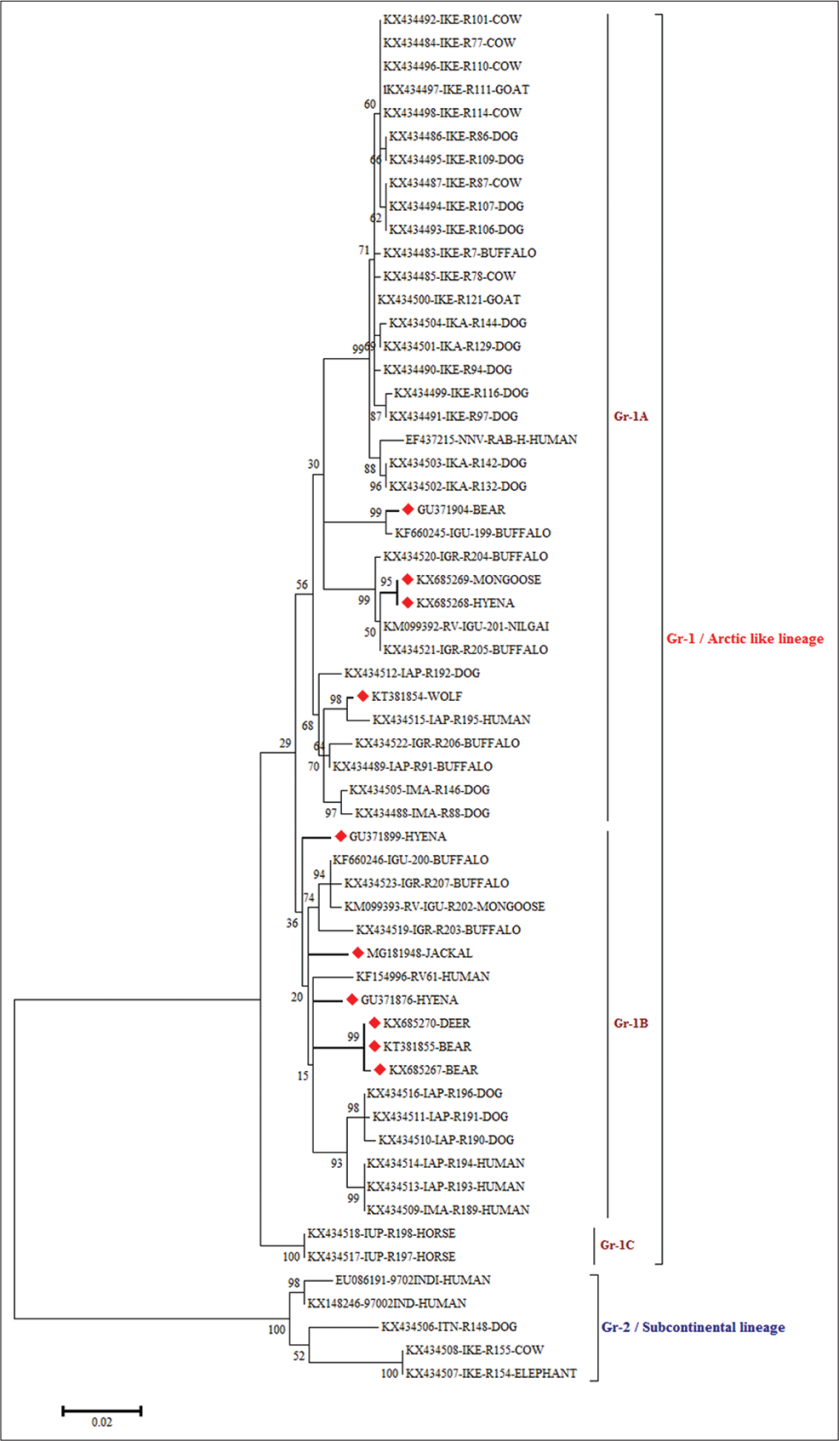
Maximum likelihood tree generated by 690 bp of nucleoprotein gene sequences showing the genetic relationship of the wild animal rabies virus isolates with other Indian rabies virus isolates. The percentage of bootstrap values given to the left of the main branch. Isolates for which the partial N gene sequence was obtained in this study are indicated by a triangle (diamond red color).

**Figure-3:**
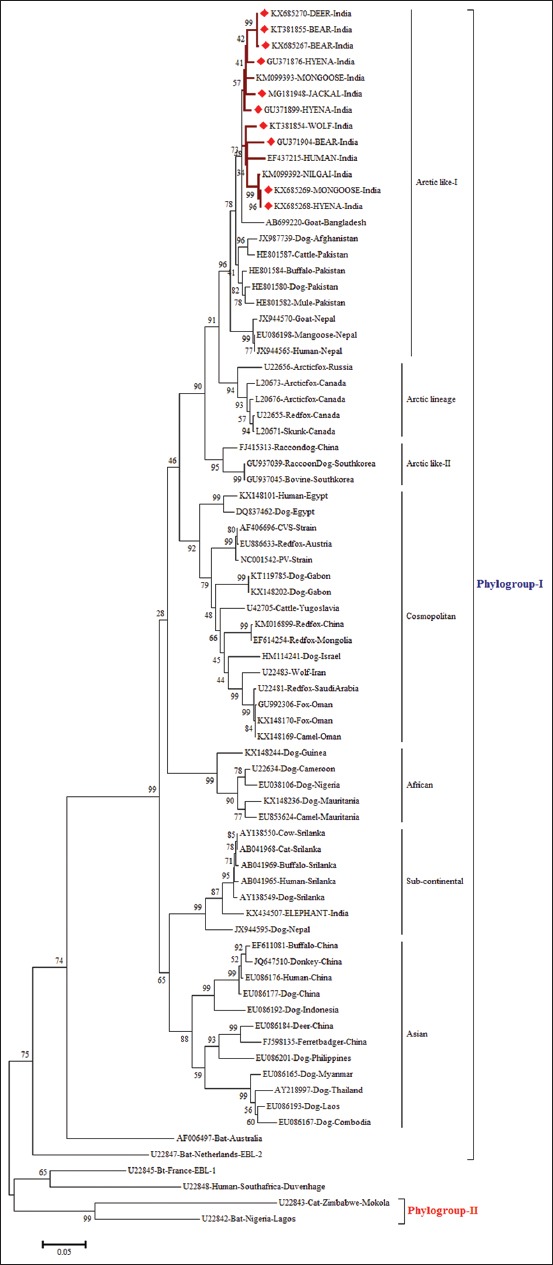
Maximum likelihood tree generated by 690 bp of nucleoprotein gene sequences showing the genetic relationship of the Indian rabies virus isolates with global RABV isolated. The percentage of bootstrap values given to the left of the main branch. Isolates for which the partial N gene sequence was obtained in this study are indicated by a triangle (diamond red color).

Of 12 nucleic acid-positive rabies samples (qRT-PCR), in 10 samples, we could get desired N gene nucleotide sequences and got accession numbers (Table-1). These 10 partial N gene nucleotide sequences were compared with published sequences of RV from different species and geographical regions of India. The phylogenetic tree revealed two distinct clades: Clade-1/Group-1 (Gr-1) or Arctic-like lineage and clade-2/Group-2 (Gr-2) or subcontinental lineage RV among the Indian RV isolates (Figure-2). The Gr-2 lineage isolates were restricted to the southern part of India, whereas Gr-1 lineage isolates were spread throughout the Indian subcontinent. Among the Gr-1, three distinct subgroups were noticed, namely Gr-1A, Gr-1B, and Gr-1C. All the RV isolates from wild animals belonged to Gr-1 RV of Indian origin (Gr-1A and Gr-1B), which are closely related to Arctic/Arctic-like single cluster and contained RV isolates from different herbivores and carnivore animals and human origin within India (Figure-2). All the wild animal RV isolates were having similar topology and grouped under Arctic/Arctic-like RV viruses (Figure-3). The wild RV isolates did not demonstrate any pattern for their host species of isolation, and even there was no pattern for geographical origin. The N gene identity of Indian wild RV isolates varied from 96% to 100%, while with Pasteur virus (PV) strain varied from 88.6% to 89.6%. The nucleotide frequencies for wild animal RV isolates were 27.64% (A), 26.18% (T), 25.62% (G), and 20.56% (C).

The N gene-derived amino acid sequence identity of wild animal RV isolates varied from 99.6% to 100%. The amino acid sequences varied at position 151 (A-G), 185 (K-R), 192 (A-T), 228 (T-A), and 231 (I-M) as compared to PV strain, whereas the majority of wild animal Indian RV isolates (WINR) showed high identity except at 185 (K-R) in WINR-5 and at 192 (T-A) in WINR-3, WINR-4, and WINR-6 compared to PV strain.

## Discussion

Till date, few studies conducted on these aspects were done with either single or two samples to best of our knowledge. The samples submitted to laboratory for confirmatory diagnosis over a period of 10 years were subjected for dFAT at first, and 12 samples were positive. Two samples were missed with dFAT compared to qRT-PCR which is attributed to the condition of samples received at the time of diagnosis as it has been reported that both environmental and non-environmental factors alter the sensitivity of dFAT [12]; especially, it is true for a country like India which is tropical in nature and less accessibility to rabies diagnostic laboratories. Even the conventional RT-PCR also showed low sensitivity than qRT-PCR which might be attributed to decomposition of samples wherein the RNases degrade the genome into smaller segments [13].

Further, degraded and lower number of nucleic acid copies might be resulted in two missed cases by RT-PCR, but which were turned-out to be positive by qRT-PCR. Three formalin-fixed brain samples of wild animals were positive for rabies by revealing characteristic round-to-oval, eosinophilic intracytoplasmic Negri bodies in the pyramidal cells of the hippocampus and cerebellar Purkinje cells. Other histopathological changes observed were neuronal necrosis, satellitosis, gliosis, neuronophagia, congestion, edema, perivascular cuffing, and meningitis [1].

The N gene-based phylogenetic tree showed two clades/groups, with Gr-2 lineage isolates which are restricted to the southern part of India, and on the contrary, Gr-1 lineage RV isolates were spread throughout Indian subcontinent. The phylogenetic analyses also showed that all RV isolates from wild animals were falling under the urban group of RV isolates from the rest of India and are closely related to Arctic/Arctic-like single cluster (Figure-3) which is an account of conservation pattern of nucleotide sequences in N gene. Among the RV strains, N gene is frequently used as a diagnostic and molecular marker for epidemiological analysis of *Lyssavirus* distribution regionally and globally compared to other genes [14]. Even though the isolates were from different geographical regions of country, they formed a single cluster which is nearer to Arctic lineage of RVs that circulate throughout the Arctic countries and are considered as phylogenetic ancestors of RV isolates in India also known as Arctic/Arctic-like lineage. The Arctic-like lineage accounts for the type circulating throughout India. Arctic RV has been shown to adapt to different hosts including fruit- and insect-eating bats and Arctic fox [15]. Our findings are in contrast to many reports of geographically restricted distribution of urban and circulation of two distinct lineages (Arctic and Asian) of RV in India [7,8]. In India, even though dog is the main reservoir of rabies transmission, the genetic similarity of the isolates from the present study reveals the possible role of wildlife in harboring RV cannot be ruled out, an issue that needs urgent attention. The unrestricted movement of domestic animals and no physical barrier between wild and domestic animals interface in India. Under these circumstances, there is a requirement of further detailed investigation of wildlife rabies cases, especially at wild-domestic-human interface and also due to a rise in wild animal transmitted rabies cases [1,2]. The close identity of RV from India with Afghanistan followed by Pakistan, Nepal, Russia, and Canada (Arctic strain) is in accordance with previous findings that the viruses from the South Asian countries and Canada have a common ancestry [7,9,15]. From this study, it can be inferred that, based on partial N gene, wild animal RV isolates are almost similar in their genetic epidemiology suggesting high endemic stability of RV in India and possibility of spillover [16]. The current study supports the spillover of long-term canine-mediated rabies enzootics into wild terrestrial carnivores complicating the control efforts and increasing the risk of exposure to humans [17]. The amino acid pattern derived from the partial nucleotide sequences of N gene did not indicate any signature substitutions with respect to the geographical origin and host species. However, previous studies on domestic animal rabies featured clustering of virus isolates according to geographical origin irrespective of host species [7,18].

## Conclusion

The partial N gene sequence analysis showed that genetically similar RV of phylogroup I is circulating both in wild and domestic animals highlighting the important role played by wild animals in the transmission and maintenance of rabies in India. Hence, there is a need for further detailed studies targeting other genes of virus or whole genome approach for elucidating the phylodynamics of rabies in wild animals. Further, understanding the epidemiology of rabies at human-wild-domestic animal interface will help in better design and implementation of rabies prevention and control in the future.

## Authors’ Contributions

Conceptualization: GBMR, RS, and KPS; Data curation and formal analysis: GBMR, SV, and BS; Funding acquisition: GBMR; Investigation: GBMR, RS, KPS, and AKS; Project administration: GBMR, RS, and KPS; Resources: GBMR and RS; Software: GBMR, SV, BS, and MS; Supervision: GBMR and RS; Validation: GBMR, RS, and KPS; Writing - original draft: MS, BS, and SV; Writing - review and editing: GBMR and MS. All authors read and approved the final manuscript.
